# 389. Trends in HIV-Related Mortality Among Reproductive-Aged Women in the United States

**DOI:** 10.1093/ofid/ofae631.124

**Published:** 2025-01-29

**Authors:** M Danial Ali Shah, Sadaf Aslam, Lauren E Rybolt, Abeer Fatima, Jacqueline E Sherbuk, Charurut Somboonwit

**Affiliations:** King Edward Medical University, Lahore, Punjab, Pakistan; University of South Florida Morsani College of Medicine, Tampa, Florida; University of South Florida, Tampa, FL; University Hospital Waterford, Waterford, Waterford, Ireland; University of South Florida, Tampa, FL; University of South Florida Morsani College of Medicine, Tampa, Florida

## Abstract

**Background:**

Despite advancements in HIV treatment, there remains a significant gap in understanding mortality rates among reproductive-aged women with HIV in the United States. To enhance evidence-based interventions, this study aims to delineate mortality trends from 1999-2020, focusing on demographic characteristics such as age, race, and geographic regions.

**Figure:**
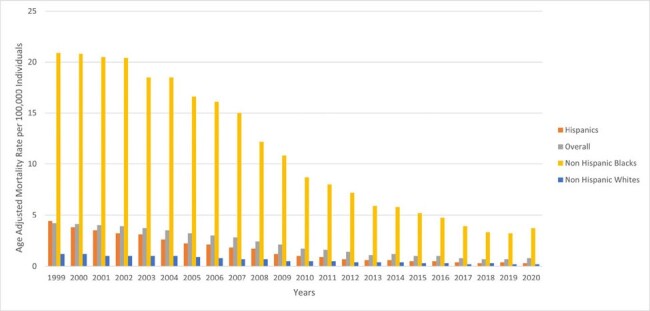

HIV-related AAMRs per 100,000 individuals stratified by race in women aged 15-44 in the United States, 1999 to 2020.

**Methods:**

This study involved a retrospective analysis using the CDC WONDER database, extracting data via ICD-10 codes B20-B24 to identify HIV-related deaths among women aged 15-44 from 1999 to 2020. We analyzed demographic disparities in HIV mortality rates over time based on age, ethnicity, and geographic regions using systematic database analysis. Results are presented as age-adjusted mortality rate (AAMR) and 95% confidence interval (CI). These were calculated by standardizing HIV-related deaths in the United States in 2000.

**Figure:**
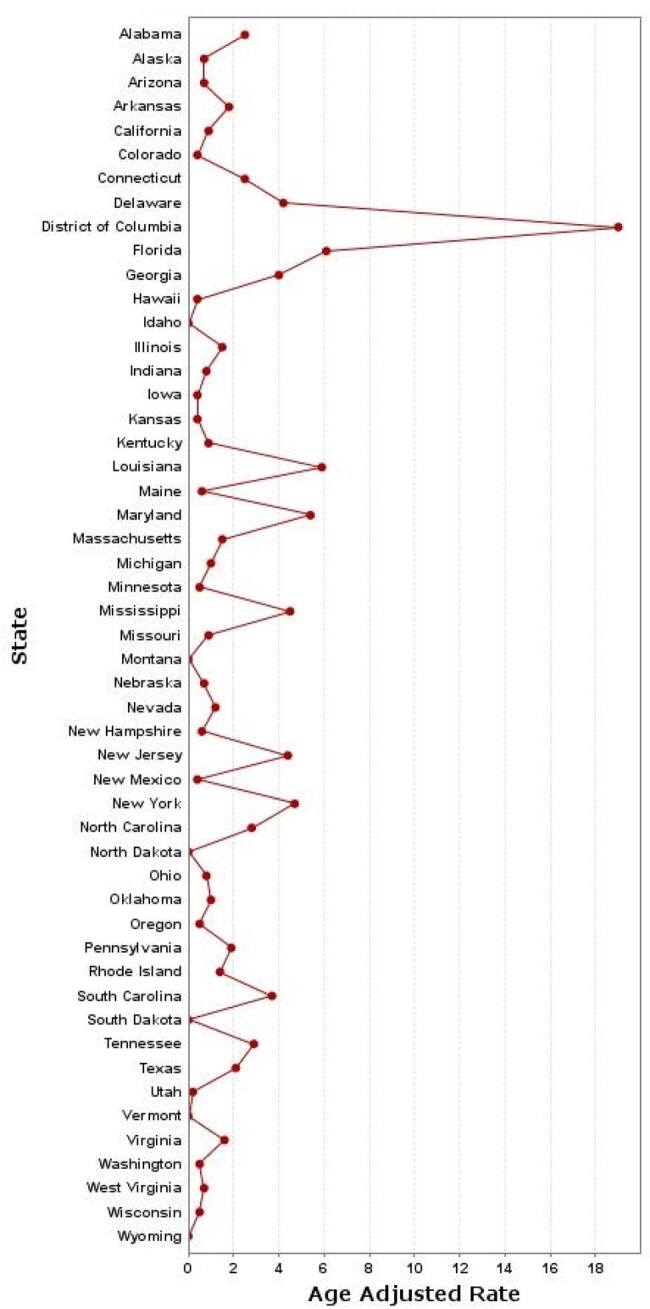

HIV-related AAMRs per 100,000 individuals stratified by state in in the United States among women aged 15-44 from 1999 to 2020.

**Results:**

Between 1999 and 2020, a total of 29,358 women aged 15-44 died from HIV in the US (AAMR = 2.2 per 100,000; 95%CI: 2.2-2.2). The crude mortality rate declined from 4.2 in 1999 to 2.1 in 2020. The overall decline in mortality rates masked disparities: Non-Hispanic Black women experienced the highest AAMR (11.3), while Hispanics, though initially higher, saw the most substantial decrease in mortality rates (1.4). Geographical analysis revealed the South as the most affected region (3.5), accounting for 58.3% of deaths, with urban areas presenting higher mortality rates than rural ones (2.4 and 1.4). Notably, older age groups (35-44) accounted for the majority of deaths (67.1%)

**Figure:**
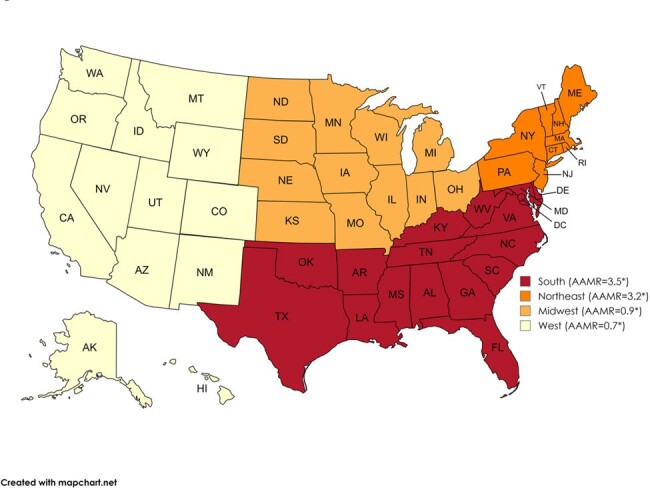

HIV-related AAMRs per 100,000 individuals among women aged 15-44 from 1999 to 2020 in the United States census regions. *=significant at p < 0.05; confidence interval does not include zero

**Conclusion:**

HIV-related mortality among reproductive-aged women decreased nationally from 1999 to 2020, yet disparities persist. Our analysis emphasizes the disproportionate impact of HIV-related deaths on non-Hispanic Black women and those residing in the Southern United States, suggesting the need for region and population-specific public health interventions. This highlights the need to improve access to care, particularly in urban areas, and target age-specific prevention programs to further reduce mortality rates. Improving timely diagnosis and addressing the stigma surrounding HIV among women is vital to reduce mortality in this demographic.

**Figure:**
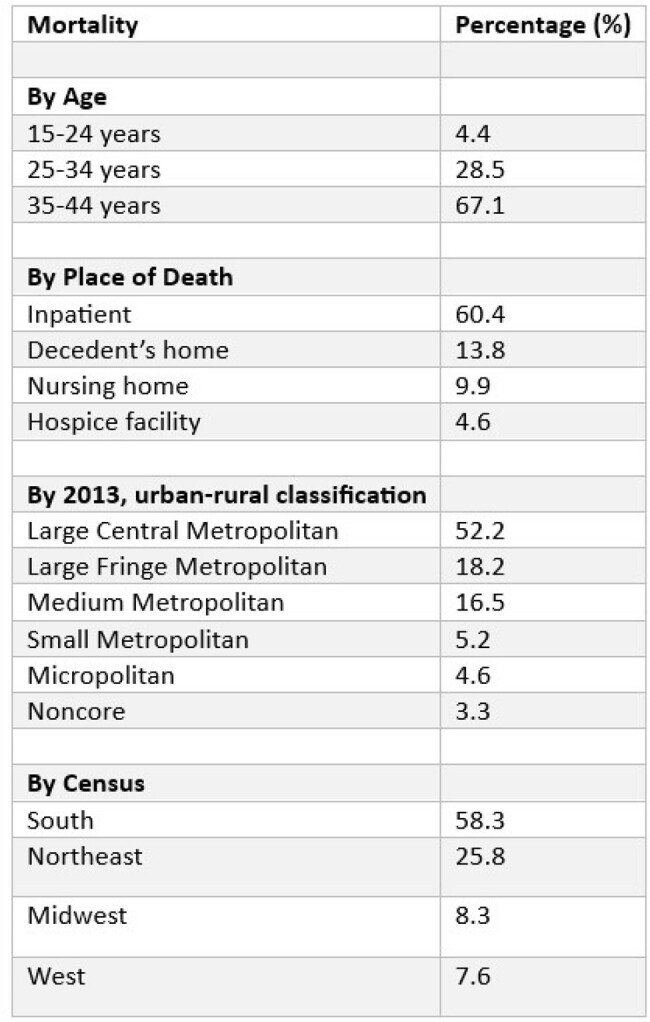

HIV-related crude mortality percentage stratified by different variables in women aged 15-44 in the United States, 1999 to 2020

**Disclosures:**

**All Authors**: No reported disclosures

